# T cells in the microenvironment of solid pediatric tumors: the case of neuroblastoma

**DOI:** 10.3389/fimmu.2025.1544137

**Published:** 2025-02-28

**Authors:** Enrico Maggi, Nadine Landolina, Enrico Munari, Francesca Romana Mariotti, Nicola Tumino, Paola Vacca, Bruno Azzarone, Lorenzo Moretta

**Affiliations:** ^1^ Tumor Immunology Unit, Bambino Gesù Children’s Hospital, IRCCS, Rome, Italy; ^2^ Department of Pathology and Diagnostics, University and Hospital Trust of Verona, Verona, Italy; ^3^ Innate Lymphoid Cells Unit, Immunology Research Area, Bambino Gesù Children’s Hospital, IRCCS, Rome, Italy

**Keywords:** immunosuppression, immunotherapy of tumors, solid pediatric tumors, T effector cells, T regulatory cells, tumor infiltrating lymphocytes

## Abstract

Neuroblastoma (NB) is an immunologically “cold” tumor with poor or no inflamed substrates as most of solid pediatric tumors (SPT). Consistent data indicate that NB tumor microenvironment (TME) is dominated by myeloid cells, with little (but variable) T cell infiltration. The obstacles to lymphocyte infiltration and to their anti-tumor activity are due to different tumor immune evasion strategies, including loss of HLA Class I molecules, high expression of immune checkpoint molecular ligands leading to exhaustion of T effector (and NK) cells, induction of T regulatory, myeloid and stromal cells and secretion of immunosuppressive mediators. In odds with adult solid tumors, NB displays weak immunogenicity caused by intrinsic low mutational burden and scant expression of neoepitopes in the context of MHC-class I antigens which, in turn, are particularly poorly expressed on NB cells, thus inducing low anti-tumor T cell responses. In addition, NB is generated from embryonal cells and is the result of transcriptional abnormalities and not of the accumulation of genetic mutations over time, thus further explaining the low immunogenicity. The poor expression of immunogenic molecules on tumor cells is associated with the high production of immunosuppressive factors which further downregulate lymphocyte infiltration and activity, thus explaining the limited efficacy of new drugs in NB, as immune checkpoint inhibitors. This review is focused on examining the role of T effector and regulatory cells infiltrating TME of NB, taking into account their repertoire, phenotype, function, plasticity and, importantly, predictive value for defining novel targets for therapy.

## 1 Introduction

Tumor-infiltrating lymphocytes (TILs) of solid tumors, and in particular T cells, influence tumorigenesis, cancer progression, metastasis, response to immune checkpoint inhibitors (ICIs) and may be predictive of clinical outcomes (1–6).

While the actual scenario of adult solid tumors highlights the coordination of circulating (cytolytic T lymphocytes -CTL, T helper -Th- and T regulatory - Treg-cells) and tissue-resident (T resident memory -Trm-, Mucosal associated invariant T – MAIT- γδ T and natural killer T-NKT-) cells (7–11), the T cell landscape of solid pediatric tumors (SPT) and in particular of Neuroblastoma (NB) is currently poorly explored and the results are often not definitive or even conflicting.

NB is the most common SPT, accounting for about 6% of all tumors in childhood, with a prevalence of 1/70,000 in children under 15yrs (12–14). Patients are usually categorized into low-, intermediate- and high-risk (LR, IR, HR) groups based on clinical stage, age at diagnosis, tumor histology, MYCN oncogene amplification and chromosomal ploidy. The great majority of LR patients with less one year displays spontaneous regression. More than 90% of LR tumors survives 5yrs or more, while only 40–50% of patients with HR NB achieves 5yrs survival: the survival outcomes drop to less than 10% for patients with relapsed metastatic disease (15–18).

Due to the poor availability of fresh tumor samples and the practical challenges of in-depth immune analyses, most studies evaluate a limited number of patients. Immunotherapy (IT) which has demonstrated successful in adult cancers, however, has low efficacy in SPT due to unsatisfactory prognostic value obtained even with new drugs (ie. anti-di-sialo-ganglioside (GD)-2 monoclonal antibody – mAb -, Dinutuximab) (12, 13). This is because NB displays low immunogenicity, due to low mutational burden and, in turn, scarce expression of neoepitopes in the context of low density (if any) of MHC-class I molecules (14, 19). Furthermore, tumor immune evasion strategies, including expression of immune checkpoint molecules, induction of immunosuppressive Treg, myeloid- and stromal cells as well as secretion of immunoregulatory factors effective, both in TME and systemically, can further impair lymphocyte infiltration and activity (20). In this review, we will analyze the features of T cells infiltrating the TME of NB, their function, plasticity, and novel targets for therapeutical interventions.

## 2 T cells in NB

Although NB has been considered an immunological “cold” tumor, the role of T cell infiltrating the TME has been recently reevaluated in several reports (21–23).

### 2.1 T effector cells

**Figure 1:**
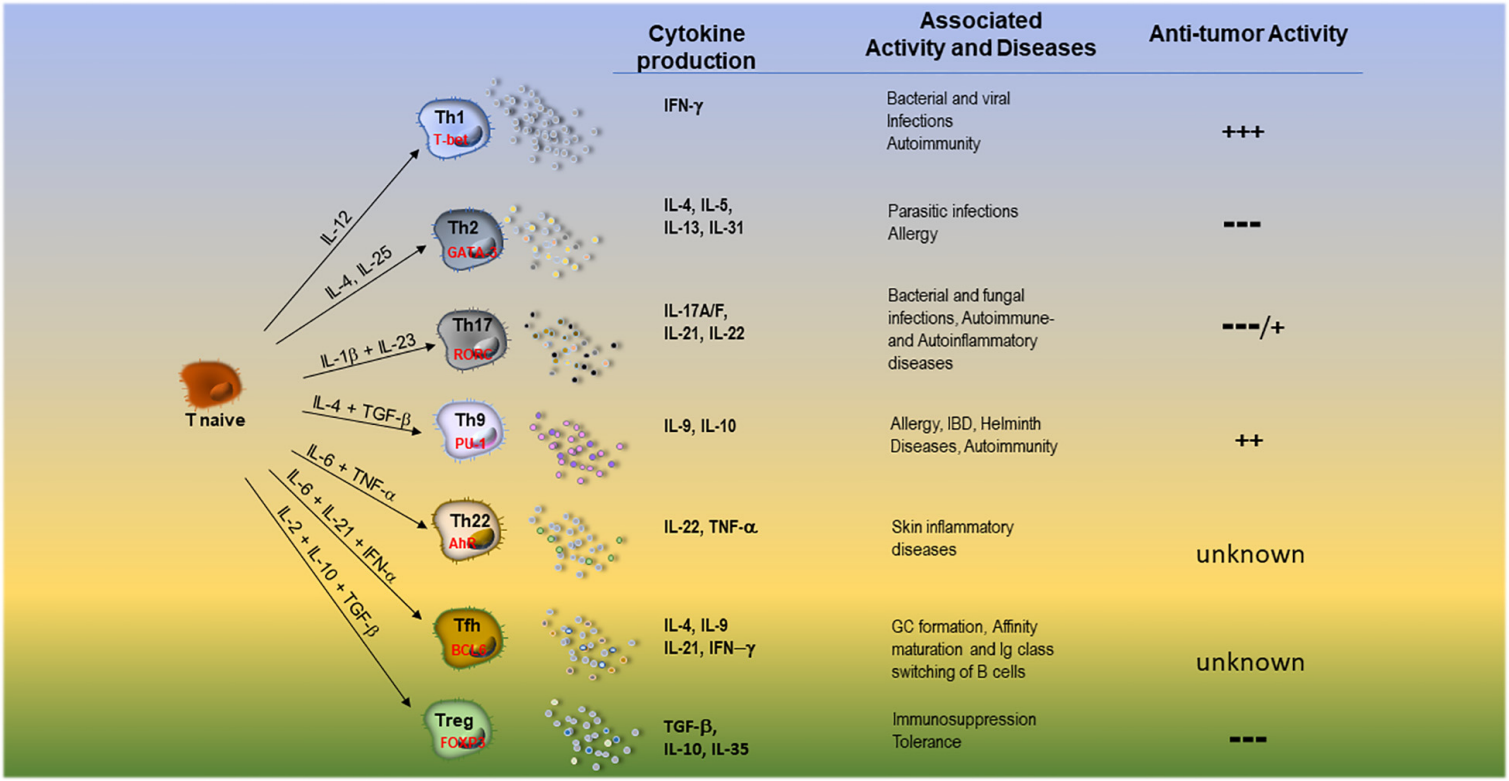
Main features of T effector cell subsets and T regulatory cells. DC-derived cytokines able to orient the development, the main transcription factors, the released groups of cytokines, the associated pathological conditions and anti-tumor activity of different T cell subsets are summarized.

The profile of immune cells infiltrating NB has been reported in few studies usually performed on a small number of specimens (24, 25). In these studies, the presence of TILs varies significantly in individual patients and tumor samples (depending on the cutoffs used): from 28% to virtually all tumors were considered positive as assessed by immunohistochemistry (IHC), even though often few lymphoid cells are detectable (26, 27). Both αβ- and γδ T cells as well as type1/2 NKT and NK cells have been identified in NB TILs, where B cells are rare or undetectable (28–33). In this regard, T cells were consistently less than 5% of total cells in the NB TME (24, 32), consisting of both CD4+ and CD8+ T cells, more frequently detectable in septa rather than in tumor cell nests (25, 34), CD8+ being predominant over CD4+T cells. Notably, recent reports utilizing computational methods identified different signatures of immune infiltration by deconvoluting bulk RNA sequencing data of tumor samples (22, 35–38). According to these studies, surprisingly, at least 50% of TME cell components were predicted to be immune cells, half of which were myeloid (DC and a small fraction of tumor- associated macrophages -TAMs-) and half lymphoid cells (37). The lymphoid compartment was mainly represented by CD4+T helper (Th) cells, while B cells and cytotoxic CD8+T cells (CTLs) were present in a small fraction, thus greatly differing from IHC-based studies (21). NKT, γδ T and NK cells were predicted to be present as well (29, 30, 36, 37, 39), while no data are available on Trm and MAIT cells. T cell infiltration has been evidenced not only in primary but also in relapsed tumors (34): a single-cell RNA sequencing (scRNA-seq) study evidenced that T cell infiltration increased in paired patient samples (diagnostic biopsy and surgical material) after neo-adjuvant chemotherapy, suggesting lymphocytes recruitment into tumor (33). Both CD4+ and CD8+ T cells showed an activated (CD25+ and HLA-DR+) profile, often with effector memory (CCR7-CD45RA-) phenotype (25). Results on freshly- or IL-2-cultured TILs from NB indicate that: i. CD4/CD8 T cell ratio ranged from 0.5 to 5; ii. rIL-2-cultured TILs show increased CD56^+^CD8^+^ T cells with cytolytic activity; iii. cultured TILs express IFN-γ, IL-4, IL-5, TNFα, IL-8, IL-10- and, to a lesser extent, IL-2 mRNAs while, in the corresponding tumor samples only TNF-α, IL-8 and IL-10- transcripts were expressed (40). Such cytokine pattern suggests a mixed CD4+ population of both Th1 and Th2 cells (31). The presence of Th17 cells in NB TILs has not been studied: only γδ T cells producing IL-17 have been described. The main characteristics of T effector/regulatory cell subsets, their master regulators, associated pathological conditions and anti-tumor activity are summarized in **Figure 1**.

### 2.2 Repertoire of T cells in NB

As previously mentioned, NB is a non-immunogenic tumor due to low/absent MHC Class I and II antigen expression and reduced adhesion molecules (41, 42). MHC-class I expression on tumor cells correlated directly with the density of tumor-infiltrating T cells (25) and with the overall survival (OS): stages 3 and 4 display lower MHC-class I expression than stages 1, 2 and 4S (25, 43). The growth of NB cells in the presence of T cells, however, suggests that these cells do not recognize and/or kill tumor cells, suggesting that T cells are not tumor antigen -TA- specific or exhausted lymphocytes. Indeed, T cells infiltrating NB are polyclonal, characterized by a diverse Vα and Vβ usage (44) and poor recognition of autologous tumor cells (ATC). However, in some patients a higher degree of limited clonality was observed in TILs (vs peripheral blood lymphocytes -PBL-), suggesting that, even though rarely, TA-specific T cell expansion may occur *in vivo*. *Ex vivo* expanded blood-derived CD8+ CTL from patients stimulated with IFN-γ-treated ATC are cytolytic for these cells, suggesting some tumor specificity (45). Anyway, the great majority of *ex vivo* expanded TILs were non-reactive to ATC, even though they maintained the capacity to home towards NB cells (46).

Importantly, the MHC-Class I low expression can be reverted by culturing NB cells with IFN-γ and TNF-α produced by activated NK cells, allowing recognition by CTLs. For instance, oncoprotein PRAME serves as immunodominant TA since NK-modulated NB cells are recognized by PRAME-specific CTL clones (47). Notably, some activated T cells can kill NB cells in an MHC-unrestricted fashion (48, 49) in a caspase-dependent- or independent manner. Soluble factors released by activated CTLs skew the phenotype of NB cells, activate caspase-8, and increase their susceptibility to lysis by TRAIL and FAS agonist antibodies (49). The recruitment of activated CTLs into the tumor may constitute a strategy for NB treatment, even in the absence of a specific TA recognition.

### 2.3 Exhaustion of T cells

T cell exhaustion occurs when cells become hyporesponsive as the consequence of persistent TCR stimulation with TA (50). The exhaustion genes resulted upregulated in NB, namely *CD244, HAVCR2, CTLA4, TIGIT* and *PDCD1* were found to be expressed in tumors with high cytotoxic signatures, indicating that chronic stimulation of T cells with their cognate antigens may lead to an exhausted state (44). Preclinical and clinical data revealed that anti-GD2 CAR-T cells may progressively lose their function *in vivo* (50). Such exhausted T cells currently express immune checkpoints (IC) as PD-1, CTLA-4, and LAG-3, thus contributing to impaired anti-tumor immune response (51–53). In this context, ICI in combination with CAR-T cells, which are beneficial in adult tumors (40, 54), display low efficacy in SPT mainly in HR NB essentially for: i. the poor or no expression of immune checkpoints on NB TME cells, ii. the effective and persistent immunosuppressive signals from the NB microenvironment on immune cells, iii.T (also CAR-T) cell exhaustion not allowing any modulation or activation with biologicals.

Other IC molecules expressed in NB are B7-H3 and CD200/CD200R. High B7-H3 expression in NB correlated with poor event-free survival (EFS), while its blockade resulted in enhanced NK cell-mediated killing of NB cells *in vitro* (55, 56). CD200 is overexpressed on NB cells and CD200^bright^ tumors have few numbers of CD4+ and CD8+ T cells producing low IFN−γ and TNF−α (57). CD200R is expressed in HLA-DR^+^CD14^+^ myeloid cells and CD11c^+^ dendritic cells (DCs), but poorly expressed in CD4+ and CD8+ T cells. Since the CD200-CD200R pathway downregulates anti-tumor immunity, also its blockade may be considered an interesting therapeutic option (57).

### 2.4 Unconventional T cells

Among lymphocytes expanded from tumor biopsies, NKT cells could play a relevant role in anti-tumor immunity by enhancing both innate and adaptive immunity. In particular CD1d-restricted iNKT (type 1 NKT) cells (58–60), characterized by Vα24-Jα18 usage, may be more abundant in LR- than HR-NB and have been reported to be predictive of good OS (61). Alpha-Galactose Ceramide (α-GalCer) stimulation induces the proliferative response of type 1 NKT cells of patients resulting in an enhancement of cytotoxicity against NB cells (40). Activated type 1 NKT (and NK) cells inhibit TAMs and MDSCs, kill tumor cells downregulating MHC antigens and secrete chemokines to recruit additional immune effectors (62).

γδT and NKT cells have also been identified in the TME of NB patients. Notably, the γδT cells (mainly those producing IL-17) seem to favor rather than impair tumor cell proliferation and migration (46).

An inverse correlation between NKT cell- and *MYCN*-mRNA is reported in primary NB tumors (30). Type 1 NKT cells migrate toward NB cells through CCL2 which is preferentially expressed by *MYCN*-non amplified (NA) tumors since active *MYCN* downregulates CCL2 (30). In agreement, type 1 NKT (and NK) cells are decreased in *MYCN*-amplified (A) tumors showing an inverse correlation between *MYCN* and NKG2D/DNAM-1 ligand expression (36, 63).

The mechanism of impaired activity of iNKT cells in NB has been recently suggested: α‐Galcer‐pulsed DCs co‐cultured with supernatants of NB cell lines are unable to stimulate iNKT cells to release IFN-γ (which is reversed by IL‐12). CD40 expression and IL‐12 release by NB supernatant-treated DCs are increased with exogenous IFN‐γ, indicating that a new type of tolerogenic DCs are active in NB TME and impair the anti-tumor effect of type 1 NKT cells (64).

### 2.5 Treg cells

Treg cells are able to inhibit the activity of T effector cells and DCs and are usually correlated with tumor progression and poor survival even though, sometimes, they can regulate TME inflammation favoring anti-tumor response. The limitation with Treg cells in NB concerns their fine detection: in humans (not in mice) Foxp3 and CD25 molecules are co-expressed in both Treg- and activated T effector cells even though this latter subset expresses Foxp3 and CD25 at low and transient level (65, 66). Since it has been demonstrated that tumor-infiltrating CD4+ T effector cells can be activated by TME signals (24), the presence of the two cell types is not an exception. This problem can explain contradictory results on the proportion and activity of TME Treg cells and their correlation with prognosis in NB. For instance, some authors report no difference of Treg cells in PB from a cohort of LR and HR patients (67), while others have shown that circulating Treg cells were higher in NB patients than in healthy children (68). Another study showed a high density of CD25+T cells in the nests and Foxp3+T cells in the septa both being associated with better survival (25). A similar problem concerns reports using scRNA-seq analysis which exclusively evaluated Foxp3 and CD25 RNA expression: in primary tumor Foxp+CD25+T cells were less expressed in HR patients and did not correlate with OS (23), whereas in BM metastases they clearly presented exhausted profile suggesting a T effector phenotype (38). Lastly and importantly, Stigliani and coworkers showed that high *Foxp3* expression of tumor biopsies predicting a better EFS and OS, was strictly correlated with perforin (PRF1) mRNA, definitively suggesting that *Foxp3* is more an indicator of activation of T effector -rather than Treg cells (61). Indeed, few studies analyze accurately Treg cell phenotype (co-expression of Foxp3, CD25^high^, CD127^low^, CD39, CD73, PD1, LAG-3, Tim3 etc.) in NB TME or evaluate their suppressive function (69, 70). One of these rare reports indicates that CD4+Foxp3+CD25^high^CD127- Treg and CD4+CD45RO+CD49b+LAG3+ T regulatory-1 (Tr1) cells in bone marrow (BM) and blood of NB patients at a lower frequency than in healthy subjects (28). However, *MYCN* amplification correlates with a higher number of BM Treg- and circulating Tr1 cells (35), even though these cells do not associate with known NB prognostic factors (28). In conclusion, the information on the presence and activity of true Treg cells in the primary and metastatic NB and the evidence of their suppressive function inside TME are currently scarce. As a consequence, studies on Treg cell repertoire in NB are completely missing.

By contrast, since in mice Foxp3 is associated exclusively to Treg cells, Treg cell depletion (Foxp3-DTR mice) in para-orthopic NB murine model, resulted in 85% reduction of tumor volume and weight and increased amounts of cytokines (IFN-γ, TNF−α, IL-4, IL-6, and IL-10) in splenocytes as compared to control mice. By contrast, tumor growth was not affected in B regulatory cell-deficient- (μMT and CD19cre) mice (71), confirming that the ablation of true Treg cells to impairing tumor growth must be considered an option for NB therapy (72).

**Table 1** summarizes the distinctive characteristics of NB subgroups according to different classifications.

**Table 1:** Immunological features of subgroups of different NB classifications.

Subgroup Classifications	Immunological Features	References
INRG		
L1, L2	Presence of CD4+ and CD8+ T cells with type1 (Th1/Tc1) and type 2 (Th2/Tc2) cell profiles	(25, 37)
M/MS	Low MHC class I on APC Low or absence of CD4+ and CD8+ T cells	(25)
RISK		
Low/Intermediate risk	Presence of CD4+ and CD8+ T cells in TME Presence of type 1 NKT cells	(25, 35, 39) (61)
High risk	Low CD4+, CD8+ T cells in TME Low type 1 NKT cells	(25, 35, 39) (61)
MYCN gene		
MYCN gene amplified	Cold tumor Low T cells with prevalence of type 2 (Th2 and Tc2) cell profile Low NK, NKT cells, monocytes and macrophages Prevalence of exhausted CTL and NK cells Low MHC class I impairing atg presentation by DCs Low CCL2 production impairing TIL homing into TME Increased PDL1 expression on NB cells Increased TME MDSC, BM Treg cells and PB Tr1 cells	(92) (28, 35, 36, 39, 44) (28, 35) (38) (39) (30, 94) (63) (35, 108)
MYCN gene not amplified	Hot tumor Presence of T (both Th1 and Th2) cells, NK, NKT cells, monocytes and macrophages, Expression of MHC-I improving atg presentation by DCs Few exhausted CTL and NK cells	(92) (28, 30, 35, 36, 39, 44) (39) (38)
HISTOLOGY		
ADRN lineage	Absence of TILs Poor immunogenic tumor cells No inflamed cells No immuno-check point expression, no response to ICIs Sensitive to differentiating/pro-apoptotic and chemotherapeutic drugs	(87) (84, 85) (82) (82)
MSC lineage	Presence of TILs with T, NK and inflamed cell infiltration Release of cytokines Immunogenic tumor cells Immuno-check point expression and response to ICIs	(87) (84–86) (82)

## 3 Impact on T cells of interactions with tumor-, stromal- and TME cells

The presence of different immunoregulatory subsets within the TME (mesenchymal stem cells -MSCs-, cancer-associated fibroblasts – CAFs-, TAMs-, tumor-associated neutrophils -TANs- and myeloid-derived suppressor cells -MDSCs-) has been correlated with unfavorable prognosis (73), conditioning the T cell infiltrate and contributing to orient the function of other TME cells. A full understanding of tumor–host interactions and T cell plasticity in “cold” tumors (as NB) will be crucial to enhance the ability of IT to attack efficiently tumor cells. Recent studies have confirmed the reduced CD4+ and CD8+ T cell infiltration in HR NB (especially in stage 4 tumors), while the higher (mainly CD4+) T cells have been associated with better OS (25, 35, 39). Some studies reported a survival benefit related to T cell infiltration also in stage 4 NB (39, 61). In this respect, Th1 cells modulate TAMs towards a pro-inflammatory antitumor phenotype (M1) while Th2 cells orient to an immunoregulatory phenotype (M2) favoring tumor progression. Whereas T cell density has a clear predictive value, the prognostic value of CD4+ or CD8+ T cells yielded conflicting results. Some reports indicated a beneficial prognostic value for CD8+ T cells (37) while CD4+ T cell prevalence occurred in tumors with unfavorable prognosis. Others highlighted that the prevalence of CD4+ over CD8+ T cells is associated with a better prognosis (25, 35). Both conditions can likely lead to favorable outcomes: the former emphasizes the role of cytotoxic cells while CD4+T cell infiltration may be crucial for the onset of an effective anti-tumor adaptive immunity. In addition, it is well known that some T effector cell subsets (such as Th2 and Th17) or Treg cells are highly plastic generating numerous transitory populations that are capable of heterogeneous cytokine release and variable cross-talk with other TME cells. As described, molecules such as the cytokine IL-12, can modulate *in vitro* Th2 or Th17 cells toward a protective Th1-oriented profile (74, 75). Treg cells also exert functional plasticity that allows them to partially acquire Th transcriptional programs referred to as Th1-, Th2-, Th17- and T follicular- Tfh-like Treg cells (76). Drugs interacting with TCR signaling, co-stimulation, and metabolic and epigenetic pathways can be used as a valid option to reprogram Treg cells toward potentially antitumor T cells. Unleashing Th programs in Treg cells, however, is not without risk, since it may upregulate auto-inflammatory and/or autoimmune mechanisms (76, 77).

The cellular and soluble signals deriving from tumor/stromal/innate cells play the major role in modifying T cell function and generating the diffuse immunosuppression of TME in NB which can explain the poor efficacy of cancer vaccines and of ICI- or CAR-T-mediated IT. Heterogenicity and immunosuppressive properties of NB cells (mainly regarding different phenotypes and *MYCN* gene amplification) and other TME components (as CAFs-, TAMs, TANs and MDSCs) and their released factors influencing the function of T effector cells will be analyzed here below.

### 3.1 T cell suppression due to NB cells

Recently, van Groningen identified two different cell types, named respectively adrenergic (ADRN) and MSC lineages within NB tumors, which correlated with the absence or presence of TILs (78–80). Specific transcriptional circuits determine the phenotype of the two subsets: while ADRN cells express markers of sympathoadrenal differentiation, MSCs are undifferentiated and similar to neural crest progenitors (78). These cellular types can spontaneously switch each other under epigenetic signals. This tumor plasticity has a major impact on cancer pathogenesis and is a potential suitable target for IT (81, 82). ScRNA-seq analysis of NB samples has also shown a subset of “transitional cells” expressing genes of both ADRN and MSC lineages (83). Each subset is characterized by distinct immune gene expression, the MSC type having the capacity of an anti-tumor immune response and ADRN lineage being less immunogenic (84, 85). MSC lineage activation promotes T cell infiltration by secreting inflammatory cytokines. These tumors are efficiently killed by CTLs and NK cells and respond to ICIs, further highlighting the role of tumor cell lineage in modulating immune response (86).

The two ADRN and MSC lineages influence the activation of innate and adaptive immune pathways (87). MSC lineage, upregulating MHC class I and promoting CTL infiltration, also favors the expression of IC ligands and exhaustion markers on T and NK cells resembling blood MSCs from healthy donors which display a clearcut suppressive activity *in vitro* (88). The presence of both immune activation and suppression markers suggests that patients with MSC-type tumors may benefit from therapy with ICIs, whereas ADRN-type tumors are more sensitive to differentiating and chemotherapeutic agents. The high expression of GD2 and anaplastic lymphoma kinase (ALK) on the two lineages can be exploited through the use of anti-GD2 antibodies plus ALK inhibitors (ALKi) (82). Notably, relapsed NB with MSC features exhibit increased immunogenicity, including elevated expression of IC ligands, suggesting that ICI may be more effective at relapse (84).

TME signals tend to force NB cells towards the ADRN-type profile (89), whereas an MSC phenotype can be acquired prevalently during therapy. Several inhibitory molecules secreted in TME compromise the anti-tumor T cell response, stimulating cancer progression, MSC-ADRN type transition and reducing the efficacy of CAR-T/NK cell therapy. Recently, another subset of tumoral neuroblasts has been identified. These so-called mature NB-MSCs display features of BM-MSCs with suppressive activity towards NK cells (90). Initially identified in cell lines (90), this subset has been recently studied on primary NB cultures (91). Our recent data indicate that the primary cultures: i. interfere with NK (and CD8+T) cell activation and function through cell-to-cell contact, ii. express high levels of GD2 independently of their phenotype, differently from NB cell lines where the MSC-ADRN-type transition associated with GD2 loss and resistance to anti-GD2 mAb treatment, iii. are resistant to NK cell killing mediated by anti-GD2 mAbs through an ADCC mechanism, iv. include undifferentiated MSCs which, in contact with NK cells, cause an immune editing phenomenon by converting these cells into mature MSCs, increasing GD2 expression (91). These data highlight the relevance of the “transitional cells” that acquire the immunoregulatory properties typical of the NB-MSCs deeply interfering with the cytotoxic activity of NK and, likely, CTLs and NKT cells.

### 3.2 T cell suppression due to MYCN gene amplification on NB cells

The *MYCN* oncogene amplification is currently considered one of the major prognostic values in NB: the TIL composition of NB differs between *MYCN*-A and *MYCN*-NA tumors, the former being “cold” and the latter “hot” tumor (92). Computational studies revealed the inverse correlation between *MYCN* amplification and leukocyte infiltration. CD8+ and CD4+ T, NK, NKT, B cells, macrophages, and monocytes, are lower in *MYCN*-A- than in *MYCN*-NA tumors (35, 36, 39, 44). ScRNA-seq of lymphocytes infiltrating untreated NB, validated by tissue microarray and TCR sequencing on NB TME, indicated that *MYCN*-NA tumors had significantly higher cytotoxic TIL signatures than *MYCN*-A tumors (28). *MYCN* silencing in NB cell lines enhances IFN-γ activity, promotes Th1-recruiting chemokines (CXCL9 and CXCL10), increases T cell infiltration (39) and expression of ligands (MICA, ULBPs and PVR) of NK cell activating receptors leading to increased cytotoxic activity (63). *MYCN* amplification impacts anti-tumor response, downregulating MHC class I antigens in NB cells and impairing TA presentation by APC leading to the decrease of anti-tumor CD8+ T cells (39). The absence of MHC class I in NB cells is responsible for the ability of *MYCN-*A tumors to escape the type 1 immune response (93). *MYCN* amplification inhibits CCL2 release which recruits TILs (30, 94). Lastly, *MYCN* activation correlates with PD-L1 expression by NB cells, favoring an immunosuppressive environment in a “hot” *MYCN*-NA tumor (63).

Activated Th2 cells have been detected in the TME of *MYCN*-A NB and positively correlated with the expression of the three hub genes (*ZNF695, CHEK1* and *C15ORF42*) proposed as potential prognostic markers and IT targets (39). Some authors established the immune gene expression profile (as *ADAM22, GAL, KLHL13*, and *TWISTT1*) in HR NB and proposed an ultra-HR NB group with the worst prognosis (95). Finally, in univariate Cox regression analysis performed in the GSE49710 dataset, two risk scores negatively associated with the presence of DCs or CD8+ T cells and, positively, with Th2 cells have been established (96).

### 3.3 T cell suppression due to CAFs

Two different clusters of CAFs have been identified in NB TME: the CAF-S1 and CAF-S4 each expressing different signatures of upregulated genes (97). Human CAF-S1 cluster strongly expresses CXCL12 and CCL2 chemokine genes (98); through CXCL12-CXCR4 interaction and CCL2 release CAFs may recruit monocytes into TME, thus favouring tumor progression, angiogenesis, metastasis, and low survival. In addition, CAFs induce monocyte differentiation into MDSCs through the IL-6/STAT3 axis (98). Likewise, signaling of the CCL2/CCR2 axis displays remarkable effects on myeloid cell survival and function and also directly enhances Treg cell suppressive activity (99). CAFs also attract Treg-cells and decrease CD8+ T cell infiltration into TME by secreting TGF-β and IL-6, likely interfering with anti-tumor immunity (100). DeClerck has isolated from primary NB tumors a cell subset of αFAP- and FSP-1-expressing CAFs sharing phenotypic and functional characteristics with BM-derived MSCs. These CAF-MSCs are classified into two different subsets on the basis of the GD2 expression. *In vivo*, the presence of the GD-2-neg subset associates with that of M2 TAMs and a type 2 immune response (101), while the GD-2-pos subset (expressed by 50% of MSCs) resulted refractory to anti-GD2 treatment (102). NB CAF-MSCs exhibit powerful immunoregulatory functions on T (103) and NK cells through the release of immunosuppressive molecules such as PGE2 and IDO-1 (104) that can be also conveyed by exosomes (105).

### 3.4 T cell suppression due to TANs, MDSCs and TAMs

A complex immunosuppressive network of potential TANs, MDSCs and TAMs has been frequently detected in human NB TME.

*TANs* are often found at the tumor site and usually promote the growth and spread of tumor cells (106) since suppress anti-tumor CTL and NK responses. Through scRNA-seq analysis two TAN subsets, named Neutrophil 1 and Neutrophil 2, expressing different gene signatures have been detected in the metastatic BM microenvironment (38). Since Neutrophil 2 express *CXCR1* and *CXCR2* they are attracted by chemokines produced by tumor cells and infiltrate the TME (107). In addition, this subset producing matrix metalloproteinases (MMP) 9 and 25 favors angiogenesis and neuroblast dissemination. Expression analysis of TAN development indicates that they are modified by tumor-released signals into immunosuppressive cells within the metastatic BM microenvironment (38).

In addition, *MDSCs* result highly positive for both the neutrophil (PMN)- and monocyte (Mo)-derived MDSC gene signature. Interestingly, the proportions of MDSCs and non-classical monocytes are higher in *MYCN*-A- compared with *MYCN*-NA tumors. Notably, a higher proportion of MDSCs has been observed in relapsed- compared to at-diagnosis tumors (108). In a murine model of NB, MDSCs *in vitro* suppressed CD8^+^ and CD4^+^ T cell proliferation whereas the same fractions from wild-type spleens had no effect. The two tumoral MDSC subsets exhibit immunosuppressive activity on T cells inducing also an exhausted state *in vivo* (98). Of note, PMN-MDSCs have been found in the blood also in different human tumors (including NB) and their number inversely correlates with disease stages and may represent an useful prognostic marker (108, 109). Importantly, it has been observed that PMN-MDSCs impair the anti-tumor efficacy of GD2-CAR T and NK cells in patients with NB (108, 109).

Different clusters of *TAMs* expressing CD68 and apolipoprotein E (APOE) have been observed in NB TME. They express a M2 signature favouring pro-tumoral activity; their number correlate with clinical stage, *MYCN* amplification, BM metastasis, histological type and risk classification. TAMs express surface toll-like receptors (TLRs) and are activated by damage- and pathogen- associated molecular patterns (DAMPs and PAMPs) derived from tumor and/or other apoptotic cells of the TME. In turn, activated TAMs secrete a large number of molecules that influence tumor, stromal and immune cells. TAMs settled near the CAF area, suggesting their close interaction within the TME (97, 110), which induces TAM polarization towards the M2/pro-tumour profile secreting CXCL1 and CXCL2 and favouring further CAFs recruitment and activation. This interaction has been associated with NB progression, chemo-resistance, *MYCN* gene amplification, and immunosuppression including Treg cells (110).

### 3.5 T cell regulatory factors released by TME cells

The interactions of monocytes with NB cells expressing MSC lineage induce the release of pro-tumorigenic- (TGF-β1, MCP-1, IL-6, IL-8, and IL-4) but not of anti-tumorigenic- (TNF-α, IL-12) cytokines/chemokines (111). Among the pro-tumor immunosuppressive molecules, TGF-*β*, migration inhibitory factor -MIF-, soluble GD2 and secreted galectin-1 have been found (35, 37, 112–114) to impair CTL and/or NK cell function (115–117). Soluble galectin-1 and MIF induce T cell apoptosis and inhibit T cell proliferation and DC maturation (37, 118, 119). High tumoral MIF is associated with low infiltration of CTLs, NKT cells, B cells and DC, and a poor prognosis in stage 4 tumors even irrespective to *MYCN* amplification (120). Tumor TGF−β is associated with shorter EFS (121); it may be produced under MSC-NB cell-monocyte interaction, inducing an amplifying loop, where TGF-β stimulates the production of IL-6 and sIL-6Rα, protecting TAMs from spontaneous death and, in turn, promoting further TGF-β production (111). Soluble GD2 can be shed by tumor cells and may be detected even at >50-fold increased concentrations in plasma particularly in patients with stage 3/4 (122, 123). In addition, it inhibits T cell proliferation (122), favoring tumor immune evasion.

Moreover, NB cells produce arginase-2, which reduces the levels of arginine (an amino acid essential for lymphocyte cell cycle) suppressing T cell proliferation *in vitro* and *in vivo*. High arginase-2 and low arginine are detected in patients with NB, both locally and systemically, and correlate with poor survival (124).

Other regulatory molecules active in NB are soluble MICA, B7-H6, HLA-G, IL-10 and high Mobility Group Box 1 (HMGB1). Soluble MICA and B7-H6 (ligands for TCR of CTLs and activating receptors of NK cells) are released from NB cells. Elevated soluble MICA downregulates its receptor (NKG2D) on NK cells, thus inhibiting the killing of tumor cells (125). Likewise, the soluble B7-H6 levels inversely correlate with the expression of activating receptor NKp30, with the occurrence of BM metastases and chemoresistance (126). Soluble HLA-G is produced by monocytes in response to NB cell exposure, inhibits CTLs and NK cells, is detectable in serum and has a prognostic value for relapse (127). HMGB1 (an inflammatory molecule modulating chromatin) is overexpressed in 11% of NB patients, associated with an elevated risk of progression, relapse and disease-related death (128). IDO-1, which converts tryptophan into kynurenines, increases NB cell resistance to the immune cells and inhibits the efficacy of GD2-CAR T/NK cells by decreasing their IFN-γ production (129). All these molecules may favor the modulation/activation/exhaustion of T cells in TME and can be responsible for the frequent inefficacy of adoptive therapies.

**Figure 2:**
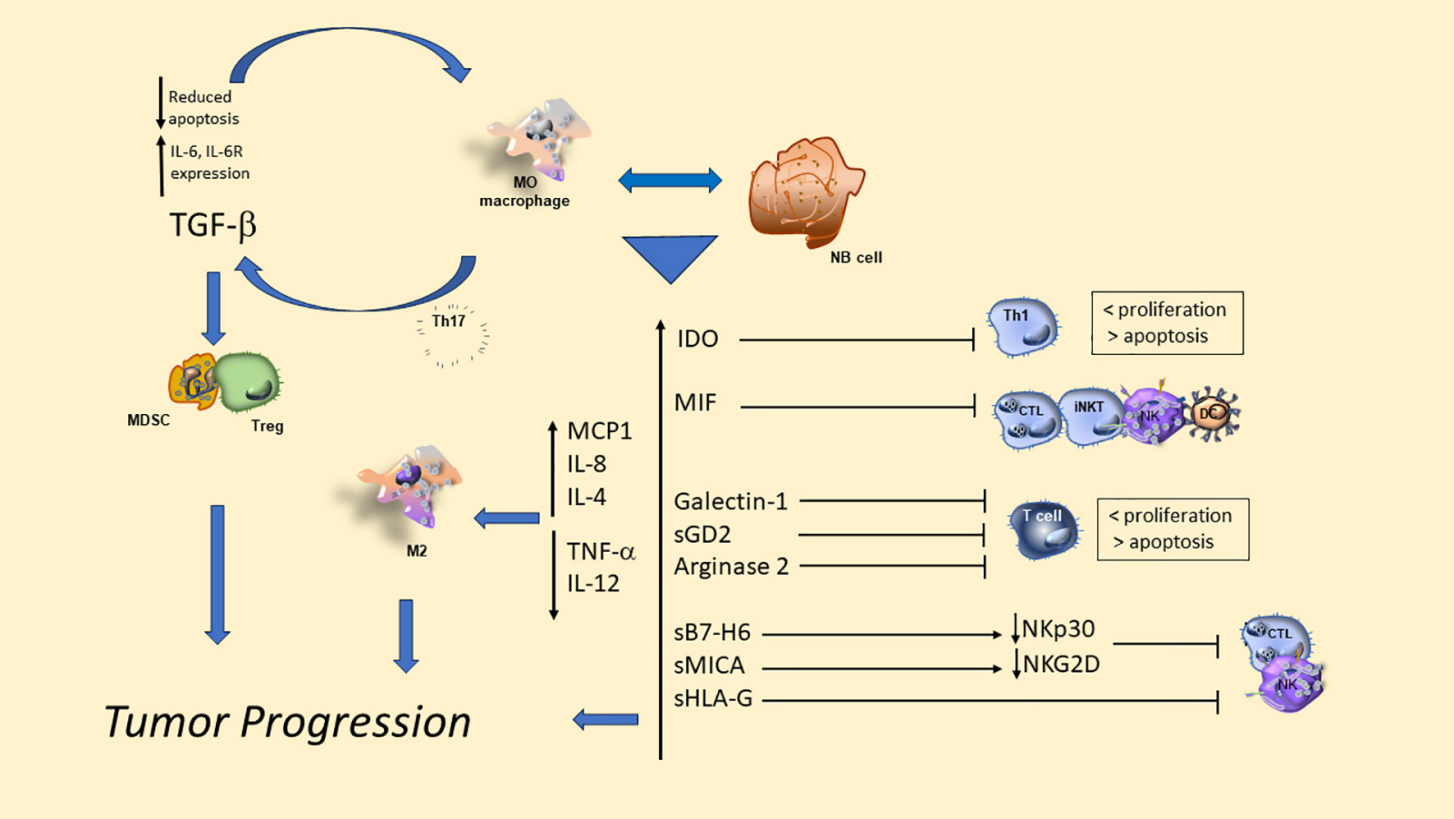
Immunosuppressive circuits between tumor cells and TAMs and their released signals in the TME of neuroblastoma reduce proliferation of T and NK cells improving their apoptosis and provide the recruitment and amplification of regulatory cells that favor tumor progression.

**Table 2** describes the new omics approaches used to characterize the NB-TME cells (130–132). **Figure 2** summarizes the signals and amplifying circuits potentially able, at least in part, to modulate TME T cells in NB.

**Table 2:** Multi-Omics profiling for NB-TME characterization.

Genomics	Genome-wide Functional screening	Epigenomics	Proteomics
Whole-genome-seq	CRISP-based functional genomic screening	ATAC-seq	Immunoassay
Immunochips		ChiP-seq	MS approaches
scDNA-seq		Dnase-seq	Cytof
		3D genome approach	Spatial proteomics

Transcriptomics	Translatomics	Spatial Transcriptomics	Metabolomics
Bulk RNA-seq	Ribosome profiling	Microdissection	MS
scRNA-seq		*In situ* hybridization	MS-based approaches
SLAM-seq		*In situ* sequencing	Glycoproteomics
snRNA-seq		*In situ* capturing	Glycomics
			Lipidomics

ATAC: ChiP: Cytof: CRISP: MS: SLAM (130–132):.

## 4 Novel targets for therapeutical interventions

Novel NB therapies involving T cells include CAR-T cells, engineered (bi- and tri-specific Immune Cell Engagers -ICEs-) and nanobodies, nanoparticles carrying drugs or mAbs, aptamers and cancer vaccines and even micro-RNA and long-term-non-coding RNA with anti-tumor activity (133, 134). Some of them are undergoing clinical trials. (https://clinicaltrials.gov NCT06528496, NCT05990751). At present engineered CAR-T cells may result the best option to threat NB. Although many preclinical studies have demonstrated the promise of this strategy (135–138), many obstacles remain. The major problems include the need to use autologous T cells, the insufficient CAR-T-cell infiltration into TME, their expansion and persistence and, importantly a reduced function in a highly suppressive TME. In this context, the PMN-MDSCs, as mentioned above, have been detected not only in the TME but also in the blood, and their number inversely correlated with the response to anti-GD2 CAR-T cell treatment (108). To overcome these barriers CAR-T cells must be balanced against potential side effects causing toxicity. New-generation CAR cell types should be correctly chosen among T, NK, γδ T, NKT cells and macrophages: however, the most promising carriers are NK cells, which do not require autologous cells, but can be donor-derived. Their storage in large numbers may be immediately available and allows to design precise quick protocols and greatly reduce the costs. Evolution of CAR-T cells should include the engineered TCR recognizing TA specific of a given tumor (or some types of tumors) and secreting T cell-engager antibodies (CAR-T.BiTE; STAb-T cells) (139–141). Moreover, to overcome the poor infiltration capability (142), the resistance to inhibitory signals (135) or T cell exhaustion blockage, dual targeting or bispecific CARs are currently developed.

Since it is presently virtually impossible to overcome all these inhibitory mechanisms, it is important to define the prevalent inhibitors of a given tumor. The results obtained with anti-PD-1/PDL-1 therapy in some tumors suggest that this approach may be successful.

**Figure 3:**
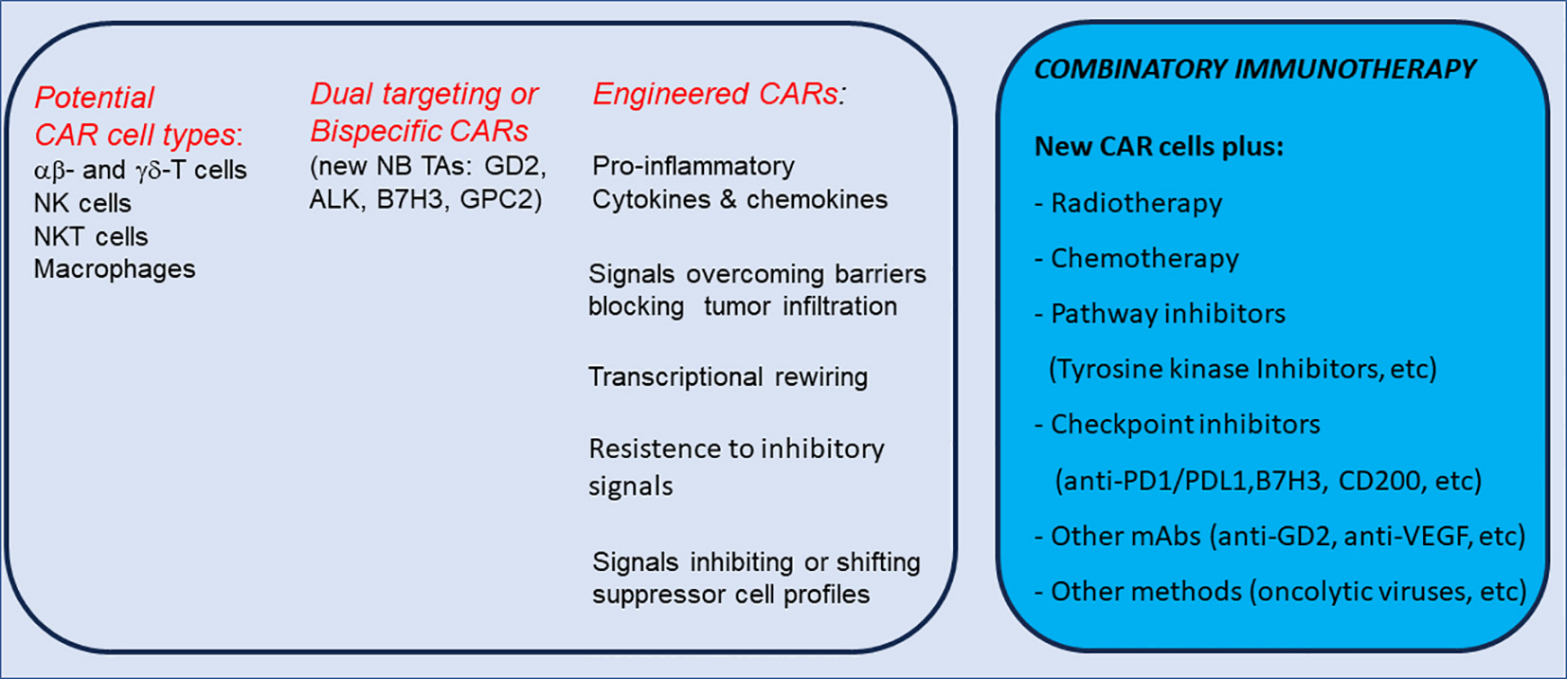
Cell types and novel technologies to enhance CAR cell specificity/sensitivity and potential combinatory immunotherapy for NB treatment.

The **Figure 3** summarizes the technologies to enhance CAR cell specificity and sensitivity for NB treatment.

Most ICEs are trans-binding bispecific antibodies (bs-Abs) consisting of two linked single-chains binding both TAs and the other cell surface trigger molecules as the activating receptors of effector cells (CD3, CD28 or 4-1BB for T cells, CD16a, NKG2D for NK cells, and CD64 for cytotoxic/phagocytic cells). Multi-specific antibodies as bs-Abs, tri-specific antibodies – ts-Abs- and even tetra-specific antibodies are currently in use in clinical trials of adults (143, 144).

As mentioned, the low number of somatic mutations in NB limits the immune responses that is induced by ICIs (145, 146). Thus, strategies of IT for NB differ significantly from those successfully used for some adult solid tumors. To date, IT is limited to target GD2, a glycolipid expressed on the surface of NB cells, which results poorly present on normal tissues. Therefore, GD2 represents the best IT target in NB. Subsequently, it has been demonstrated that some developmental proteins have been found to be highly expressed in NB cells but not in healthy tissues, thus serving as unique and specific targets for IT. These targets have been identified and include GPC2, B7-H3, and ALK.As discussed above, the NB TME prevents the infiltration of endogenous immune cells since it contains suppressor cells as type 2 TAMs, MDSCs and Treg cells interfering with immunotherapeutic strategies. Therapies as radio- or chemo-therapy, or CD47 “don’t eat me” signal blockade on tumor cells focused to reverse suppressive microenvironment, may improve the antitumor efficacy of endogenous immune cells or of genetically modified CAR-T cells. Indeed, radiotherapy can influence the suppressive NB TME turning cold- into hot tumors essentially by spreading: i. DAMPs from irradiated cancer cells promoting DC processing and presentation of TAs to T cells ii. cytoplasmic dsDNA which are accumulated after radiation, activating the cGAS-STING and TLR9 pathways and leading to type I IFN release and DC maturation (147). On the other hand, several chemotherapeutic drugs used in NB treatment inhibit Treg cell proliferation and induce MDSC lysis in TME in addition to triggering an immunogenic cell death pathway, which stimulates robust innate and adaptive immune response by exposing DC to TAs and DAMPs (148–150).Thus, a chemo-IT approach combining cytotoxic agents with anti-GD2 mAbs has resulted successful for several NB patients and is currently considered the standard of care for children with relapsed or refractory disease (151–154).

Additional combinations of IT with traditional cytotoxic agents and radiotherapy, small-molecule inhibitors of oncogenic pathways, and IT should be carefully considered in the future. Since next-generation small molecules, as ALK-, aurora-A- and CDK9/2 inhibitors (155–157), are being integrated into treatment for appropriate patients, their ability to really synergize with combination IT regimens will require careful analyses in precisely defined pre-clinical- and clinical studies (135, 142, 158).

**Figure 4:**
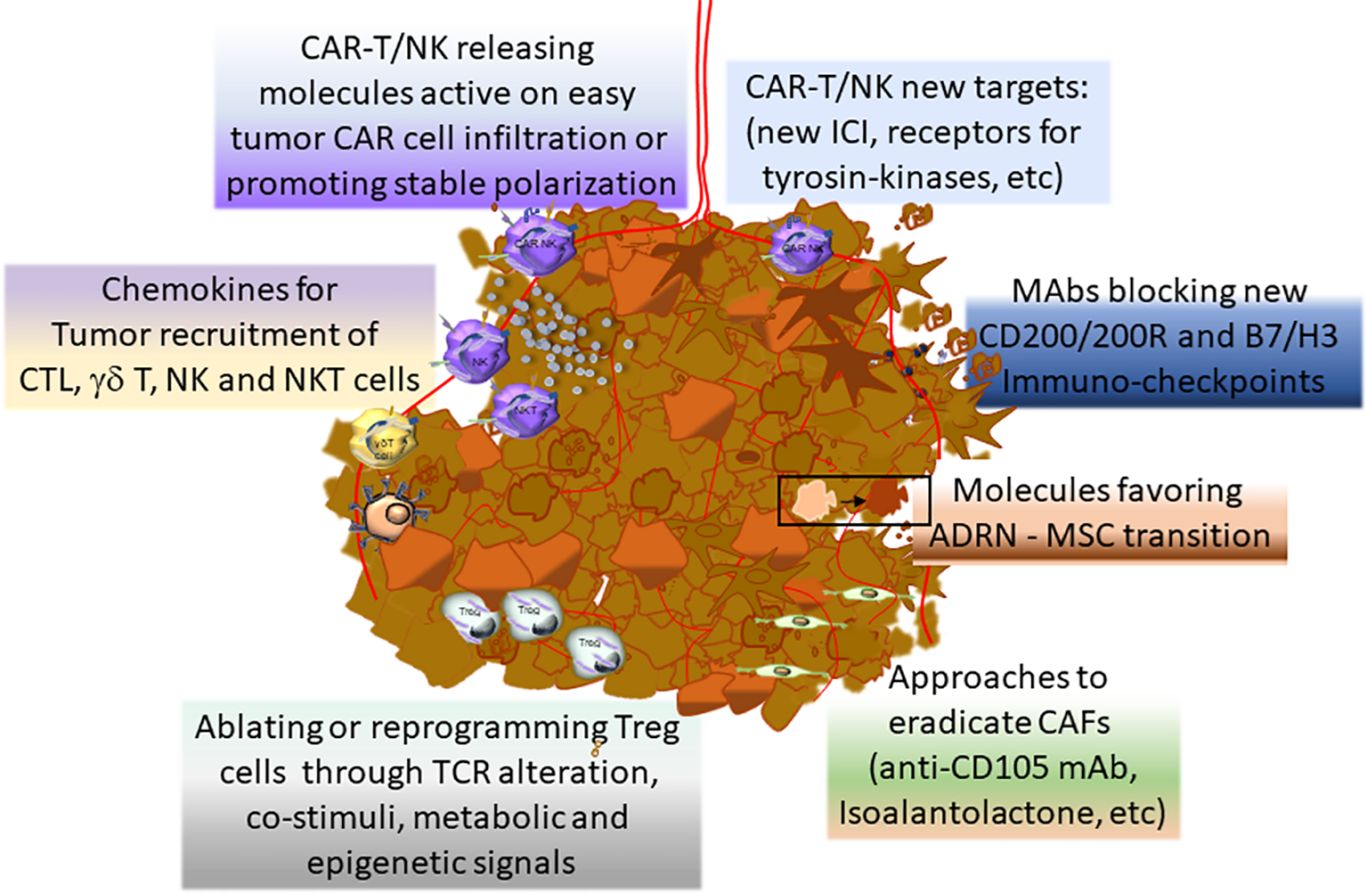
Novel therapeutical approaches to improve the efficacy of NB treatment.

**Figure 4** summarizes the main novel therapeutical approaches for NB.

## 5 Conclusions

The limited efficacy of current IT, CAR-T/NK therapy or cancer vaccines is the hallmark of the majority of solid pediatric tumors, including NB. While remaining potentially the most effective therapy, several problems on adoptive therapy of NB remain open, mostly those causing low efficacy of CAR-T cells. The ability of TCRs to target intracellular proteins (as oncoproteins) offers a valid opportunity to treat NB. This requires the need for CAR-T cells: i. to traffic easily to tumor sites, ii. to cross through tumor endothelial and stromal barriers iii. to recognize and kill efficiently cancer cells, iv. to survive and maintain their activity in a highly immunosuppressive environment.

The difficulty of lymphocyte infiltration and of their sufficient expansion *in vitro* for a possible adoptive treatment (159, 160) are due to some evasion strategies of NB, as expression of IC, induction of different immunosuppressive myeloid and stromal cells and secretion of immunoregulatory molecules leading to poor TIL infiltration and deficient/inadequate anti-tumor reactivity (20). The last Pediatric Strategy Forum of the Consortium of European Society for Pediatric Oncology and Innovative Therapies for Children with Cancer in Europe, established that the lack of knowledge on the immunological landscape of SPT is responsible for the failure of IT assayed so far (161). For instance, the eradication of CAFs with anti-CD105 mAbs-mediated ADCC or the use of drugs such as iso-alantolactone able to induce aging and functional inactivation of CAFs (162), could lead to the loss of M2 macrophages and MDSCs and, therefore to efficient CTL recovery. Finally, the development of CAR-T cells engineered to secrete proinflammatory cytokines (such as IL-12, IL-15 and IL-18), to produce molecules engaging tumor cells by not engineered T cells (162–166), as well as the development of strategies to overcome cell exhaustion could also be required (140, 167, 168).
